# Chemometrics in Europe: Selected Results

**DOI:** 10.6028/jres.093.036

**Published:** 1988-06-01

**Authors:** Wolfhard Wegscheider

**Affiliations:** Institute for Analytical Chemistry, Mikro- and Radiochemistry, Technical University Graz, A-8010 Graz, Austria

Chemometrics is a very international branch of science, perhaps more so than chemistry at large, and it is therefore appropriate to question the suitability of the topic to be presented. It is, however, the author’s opinion that the profile of European chemometric research has a couple of distinct features that may originate more in the structure of the educational system than in the actual research topics. The profile as it will be presented is the one perceived by the author, and therefore comprises a very subjective selection of individual contributions to the field. Obviously, this is not the place to offer a review on chemometrics, let alone one that is restricted to a continent.

The definition of chemometrics [1] comprises three distinct areas characterized by the key words “optimal measurements,” “maximum chemical information” and, for analytical chemistry something that sounds like the synopsis of the other two: “optimal way [to obtain] relevant information.”

## Information Theory

Eckschlager and Stepanek [2–5] pioneered the adaption and application of information theory in analytical chemistry. One of their important results gives the information gain of a quantitative determination [5]

$$\hat{I}\left(q\|p\right)=\ln\frac{\left(x_{2}-x_{1}\right)\sqrt{n_{A}}}{s\sqrt{2\pi e}}$$ (1)

where *q* and *p* are the prior and posterior distribution of the analyte concentration for the specific cases of a rectangular prior distribution in (*x*_1_, *x*_2_) and a Gaussian posterior with a standard deviation *s* determined from *n*_A_ independent results. The penalty for an inaccurate analysis is considerable and can be expressed as

$$\hat{I}\left(r;q,p\right)=\hat{I}\left(q\|p\right)-\frac{n_{A}}{2}{\left(\frac{d}{s}\right)}^{2}$$ (2)

with *d* the difference between obtained value and the true value of *x*. The concept has also been extended to multicomponent analysis and multi-method characterization. In the latter case, correlations between the information provided by the different methods need to be accounted for. Given the cost of and time needed for an analysis, *information efficiency* can be deduced in a straightforward manner [2]. Recently, work was published [5] suggesting the incorporation of various relevance coefficients; this, indeed, is a very important step since it provides a way to single out the information that is judged to be relevant for a given problem. It also opens up the possibility to draw on information theory for defining objective functions in computer-aided optimization of laboratory procedures and instruments.

## Optimization of Chromatographic Separations

In the area of optimization, significant activity is spotted in chromatography since systematic strategies for tuning the selectivity are badly needed. Due to the randomness of elution, a hypothesis supported recently by Martin et al. [6], peak overlap is the rule rather than the exception even for systems with high peak capacity. Assuming equiprobable elution volumes for all analytes, the authors [6] have derived from standard probability theory an expression for the probability that all analytes are separated. An evaluation of eq (9) in reference [6]

$$P_{m,n}\left(m\right)={\left(1-\frac{m-1}{n-1}\right)}^{m-2}$$ (3)

(*m* the number of components, *n* the peak capacity of the system and *P_m,n_*(*m*) the probability that all components are separated) is provided in table 1 and shows that the absence of overlap is really very improbable except in cases of an extremely high ratio of peak capacity/number of components. The necessity of a separate optimization for each mixture of compounds is thus the rule and not an exception.

**Figure 1:**
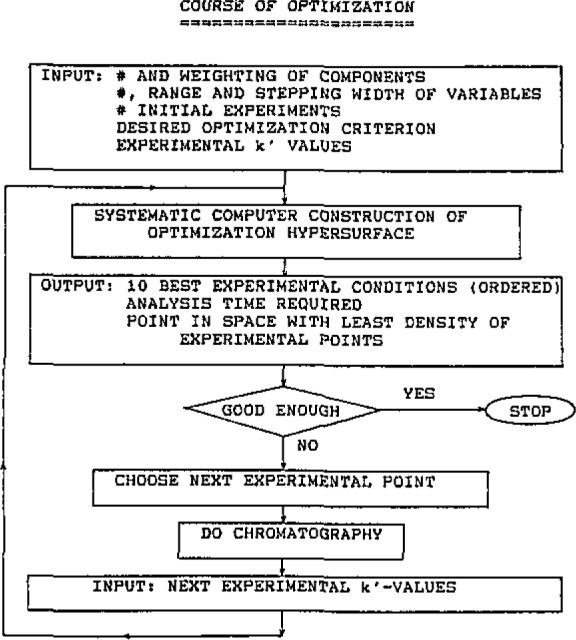
Flow chart for using the “moving least-squares” algorithm [8].

Chromatographic optimization cannot be reliably accomplished, however, by direct search techniques like the SIMPLEX, since each change of elution order corresponds to a minimum in the objective function. For multimodal surfaces, techniques that model the retention behaviour of each analyte separately and subsequently assemble a response function for a grid-like pattern in the space of experimental variables seem to be most promising [7]. Lankmayr and Wegscheider [8] have suggested to use a “moving least-squares” algorithm known from the interpolation part of the X-ray fluorescence program NRLXRF [9] for modelling elution. This results in an efficient optimization that is not stranded on secondary maxima of the response surface. A flow chart of this procedure is provided in figure 1. The efficiency stems mainly from three things: (i) a first estimate of the optimum can be made from only two chromatographic runs under different conditions; (ii) additional data can be incorporated to update the model of the response surface as they become available; and (iii) if required, additional experimental variables can be entered in the optimization at any stage. A drawback of this and all similar approaches is that an assignment of the peaks to the analytes is required at each stage. Currently, experimental work is under way to evaluate fuzzy peak tracking based on peak area and mean elution order [10].

## Selectivity and Multicomponent Analysis

In spite of the great advances in the development of methods at trace levels, neither current separation techniques nor the detection principles are sufficiently selective. An attempt was recently made to redefine selectivity in chemometric terms, since Kaiser’s well received proposal on this subject fails in two ways [11]. First, it does not give a clear account of the interrelationship between selectivity, accuracy and precision, and second it gives a value of infinity for full selectivity, a somewhat fuzzy result. By Taylor expansion, any multicomponent calibration system can be assessed for its selectivity by the condition number of the sensitivity matrix, cond(*K*); the condition number assumes the value of one for full selectivity and grows as the mutual dependence of the columns of *K* increases. Since the condition number can also be shown to relate to the analytical error, it was termed “error amplification” factor [12]. It can thus be regarded a prime concern in multicomponent analysis to find suitable means to minimize the condition number.

Recently, a chemometric approach was suggested by Lohninger and Varmuza [13], who derived a linear discriminator to serve as a selective detector for PAH in GC-MS. This was accomplished by selecting the best of 40 proposed features both in terms of discriminatory power against NOT-PAHs and by a rigorous sensitivity analysis to discard those that are unreliable in the presence of experimental error. The authors claim a recognition rate of 99%. Surely the concept is in its infancy, but the chemometric detector may well have potential in other areas, as well.

## Partial Least Squares and Multivariate Design

Relating sets of measurements to each other is frequently done by least squares techniques. Among those, the partial least squares (PLS) algorithm as developed by Wold et al. [14] is most popular among chemometricians. As opposed to principal component regression, another remedy in case of collinearity, PLS enhances relatively small variance structures if they are relevant for predictions of *Y* from *X.* This is done by using eigenvectors of *X′YY′X* instead of *X′X* as done in principal components analysis [15]. The most recent application of PLS are in quantitative structure activity relation (QSAR) studies of various classes of compounds.

Examples are contained in reference [16] and briefly outlined in reference [17]. For a series of bradykinin—potentiating pentapeptides, a model was derived to predict the biological activity of another set of peptides by describing the amino acid sequence from principal properties of single amino acids. These principal properties were deduced from 29 parameters by principal components analysis. These same principal properties can be used to design an approximate Fractional factorial function with 16 different structures that effectively spans the space in terms of the 29 parameters initially judged useful for the description of the relation between structure and biological activity. This gives a guideline for the synthesis of good candidate peptides. As pointed out by Wold and coworkers [17], current advice in peptide design is to change the amino acid in only one position at a time, in clear contrast to what has been shown to be efficient screening of design variables.

## Fuzzy Theory in Analytical Chemistry

An important strength of chemometrics as applied in analytical chemistry must be to utilize the wealth of statistical, numerical, computational methods available and choose the appropriate one in each instance. Recently, fuzzy theory was introduced in analytical chemistry [18]. Its basic notion is that crisp sets and numbers are replaced by their fuzzy analogues, to allow for variability on a nonprobabilistic basis. The type of variability is defined in a so-called membership function that can be constructed from supplemental knowledge or subjective judgement by an expert in the field. By doing this, each number manipulated also carries information on its spread. Important applications of fuzzy theory so far have been library searching [19], pattern classification [20], and calibration with linear and non-linear signal/analyte dependencies in the presence of errors in *x* and *y* [21]. In the future, the incorporation of fuzzy information in expert systems will undoubtedly play a major role.

## Conclusions

As pointed out at the beginning, this is only a subset in terms of volume of work done in chemometrics in Europe today. It is hoped that the reader finds this useful since it is regarded as typical. Other papers in this volume will cover calibration [22], evolving factor analysis [23], and expert systems in analytical chemistry [24], all being subjects of intense study in European laboratories. More traditional fields, for example, cluster analysis and pattern recognition, although very actively pursued, have not been included in this abstract [25].

## Figures and Tables

**Table 1:** Probability that all components in a mixture are separated

Peak capacity number of components probability		
50	5	0.77
	10	0.20
	20	1E-4
100	5	0.88
	20	0.02
	50	5E-15
1000	5	0.99
	50	0.09
	100	4E-5
